# Supplementary material for: Formation of extraterrestrial peptides and their derivatives
Source: Sci Adv. 2024 Apr 17;10(16):eadj7179. doi: 10.1126/sciadv.adj7179 (PMC11023503; doi:10.1126/sciadv.adj7179)
Supplement: Supplementary file 1 — Figs. S1 to S6 Tables S1 and S2 [file sciadv.adj7179_sm.pdf]

Science Advances
AAAS

Supplementary Materials for

Formation of extraterrestrial peptides and their derivatives

Serge A. Krasnokutski *et al.*

Corresponding author: Serge A. Krasnokutski, sergiy.krasnokutskiy@uni-jena.de;
Pauline Poinot, pauline.poinot@univ-poitiers.fr

Sci. Adv. **10**, eadj7179 (2024)
DOI: 10.1126/sciadv.adj7179

This PDF file includes:

Figs. S1 to S6
Tables S1 and S2

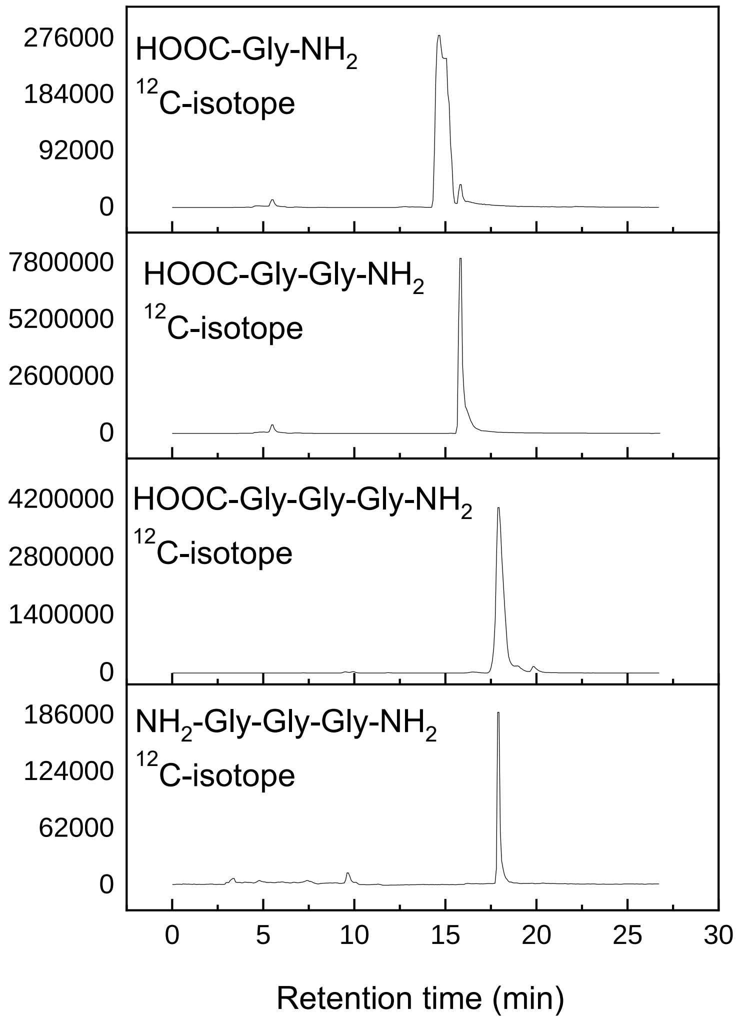

Fig. S1.

Ion signal on the masses of the specified molecules as a function of the retention time from ultra-high-performance liquid chromatography (UHPLC) analysis of the chemical standards purchased commercially.

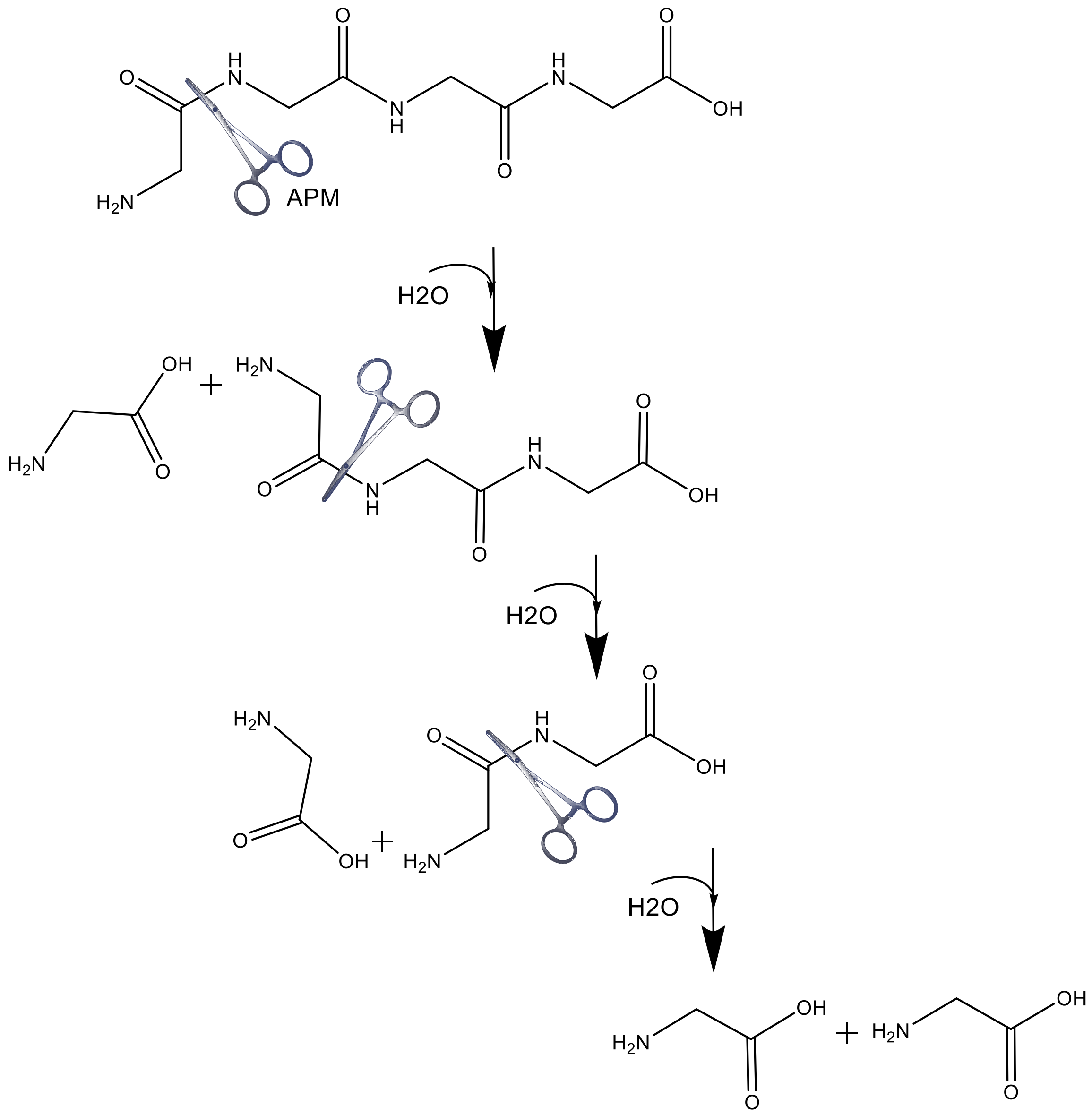

Fig. S2.

Schematic diagram of APM function leading to the formation of glycine.

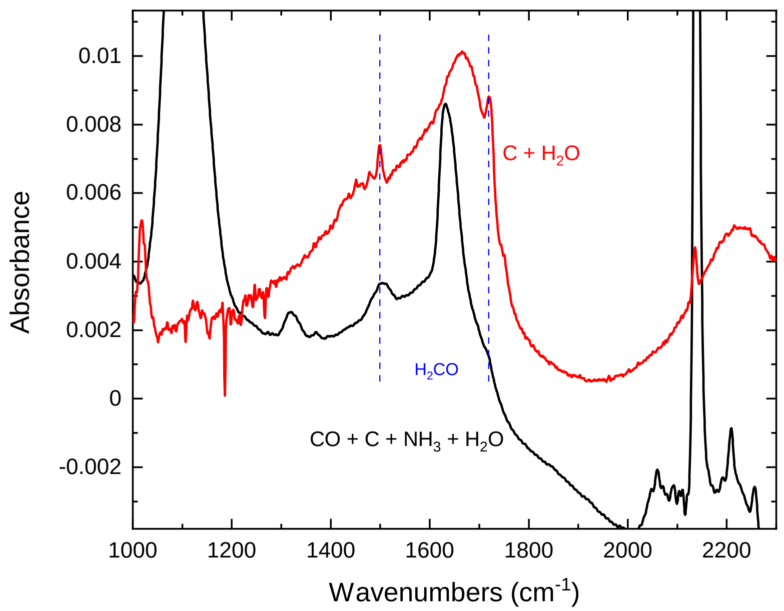

Fig. S3.

The IR absorption spectra of ice produced at 10 K on the substrate after the codeposition of the specified reactants. The vertical dashed lines denote the position of the absorption bands of formaldehyde (H_2CO).

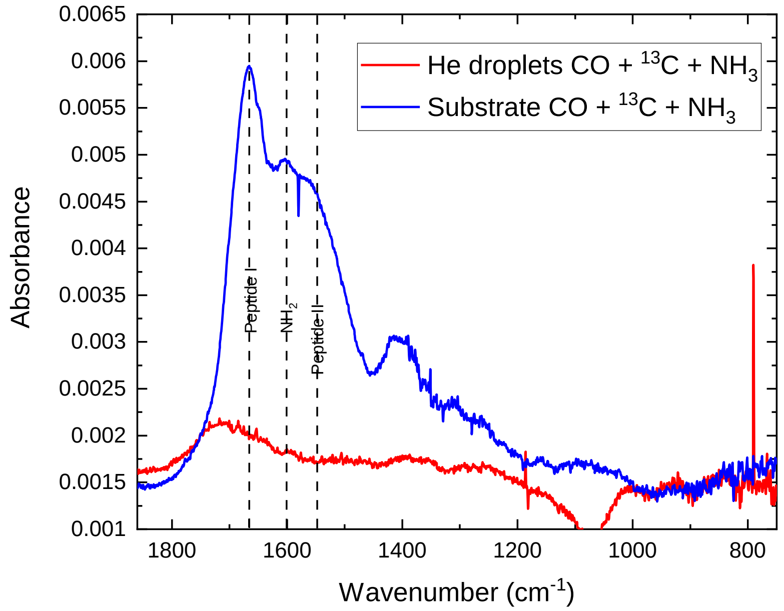

Fig. S4.

Comparison of the IR absorption spectra of RTR material produced after depositing reactants on the substrate kept at 10K and inside of He droplets according to the scheme depicted in Fig S4.

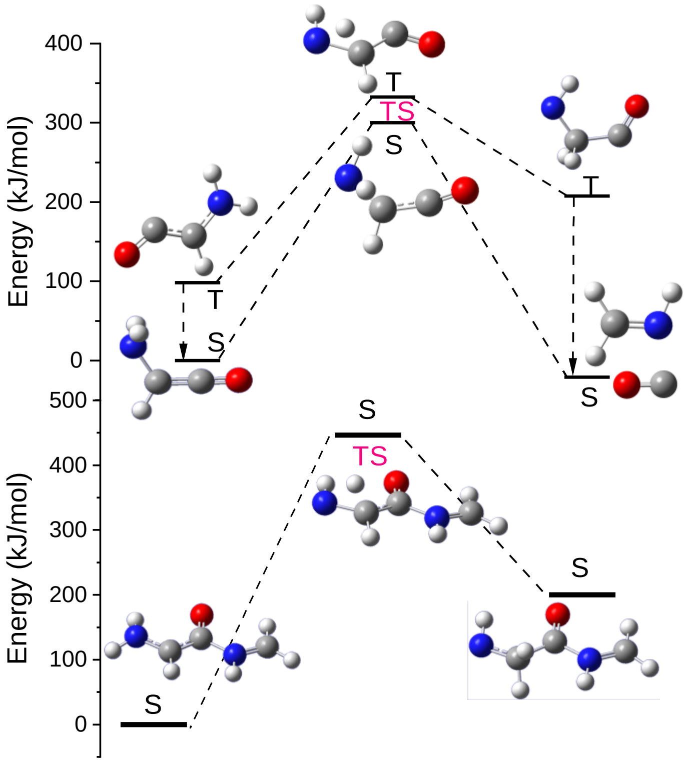

Fig. S5.

Energy level diagram for the intramolecular proton transfer from NH_2 group to alpha carbon in aminoketene and in fragmented glycine dipeptide which is formed after intramolecular proton transfer. T and S denote the triplet and singlet states correspondingly and TS stands for the transition states.

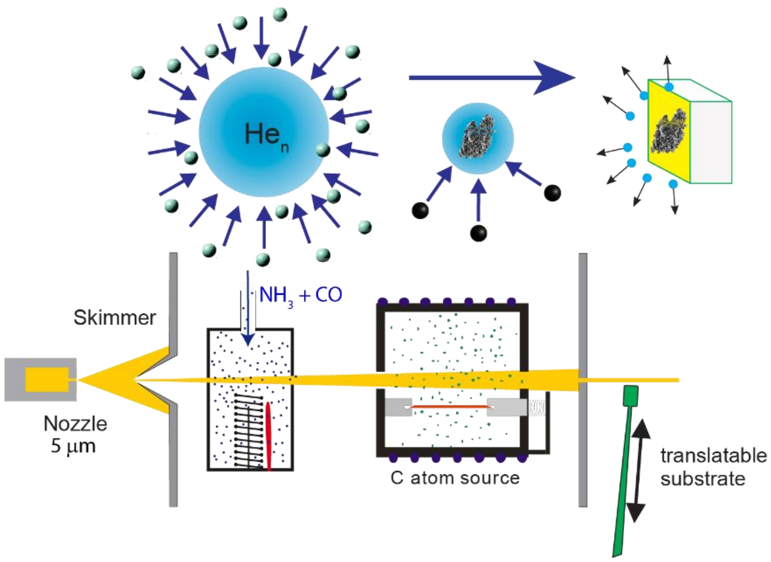

Fig. S6.

Principle scheme of the experiments with He droplets.

Table S1.

UHPLC-HRMS ions extraction: ^{13}C- and ^{12}C-compounds detection

Molecules	Formula	Exact Mass	UPLC-HRMS Detection
HOOC-Gly-NH_2 ^{12}C-isotope	$C_2H_5N_1O_2$	$[M+H]^+$ = 76.0393	X
HOOC-Gly-NH_2 ^{13}C-isotope	$C_1{}^{13}C_1H_5N_1O_2$	$[M+H]^+$ = 77.0426	X
HOOC-Gly-Gly-NH_2 ^{12}C-isotope	$C_4H_8N_2O_3$	$[M+H]^+$ = 133.0608	X
HOOC-Gly-Gly-NH_2 ^{13}C-isotope	$C_2{}^{13}C_2H_8N_2O_3$	$[M+H]^+$ = 135.0675	X
H_2N-Gly-Gly-Gly-NH_2 ^{12}C-isotope	$C_6H_{11}N_3O_4$	$[M+H]^+$ = 190.0822	X
HOOC-Gly-Gly-Gly-NH_2 ^{13}C-isotope	$C_3{}^{13}C_3H_{11}N_3O_4$	$[M+H]^+$ = 193.0923	X
H_2N-Gly-NH_2 ^{12}C-isotope	$C_2H_6N_2O_1$	$[M+H]^+$ = 75.0553	X
H_2N-Gly-NH_2 ^{13}C-isotope	$C_1{}^{13}C_1H_6N_2O_1$	$[M+H]^+$ = 76.0586	√
H_2N-Gly-Gly-NH_2 ^{12}C-isotope	$C_4H_9N_3O_2$	$[M+H]^+$ = 132.0767	X
H_2N-Gly-Gly-NH_2 ^{13}C-isotope	$C_2{}^{13}C_2H_9N_3O_2$	$[M+H]^+$ = 134.0835	√
H_2N-Gly-Gly-Gly-NH_2 ^{12}C-isotope	$C_6H_{12}N_4O_3$	$[M+H]^+$ = 189.0982	X
H_2N-Gly-Gly-Gly-NH_2 ^{13}C-isotope	$C_3{}^{13}C_3H_{12}N_4O_3$	$[M+H]^+$ = 192.1083	√

✓: Detected; ×: Not detected

Table S2.

MRM transitions and MS parameters. In this analysis, the electrospray ionization produced positively charged ions.

Molecules	Formulae	Structure	MRM transitions Precursor ion > Fragment ion	Collision energy (eV)	Dwell time (ms)
HOOC-Gly-NH_2 ^{12}C-isotope	$C_2H_5N_1O_2$	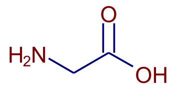	76.30>30.15<br>76.30>31.10	12<br>33	10<br>10
HOOC-Gly-NH_2 ^{13}C-isotope	$C_1{}^{13}C_1H_5N_1O_2$	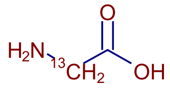	77.05>31.05<br>77.05>32.05	12<br>33	10<br>10
HOOC-Gly-Gly-NH_2 ^{12}C-isotope	$C_4H_8N_2O_3$	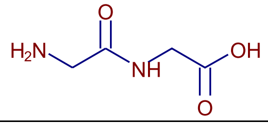	133.35>76.15<br>133.35>30.20	12<br>26	10<br>10
HOOC-Gly-Gly-NH_2 ^{13}C-isotope	$C_2{}^{13}C_2H_8N_2O_3$	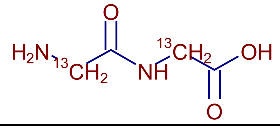	135.05>77.05<br>135.05>31.05	12<br>26	10<br>10
H_2N-Gly-Gly-Gly-NH_2 ^{12}C-isotope	$C_6H_{11}N_3O_4$	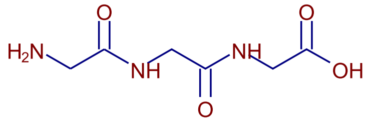	190.30>115.20<br>190.30>87.10<br>190.30>76.10	11<br>14<br>20	10<br>10<br>10
HOOC-Gly-Gly-Gly-NH_2 ^{13}C-isotope	$C_3{}^{13}C_3H_{11}N_3O_4$	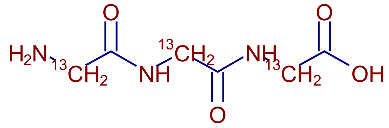	193.10>117.05<br>193.10>89.05<br>193.10>77.05	11<br>14<br>20	10<br>10<br>10
H_2N-Gly-NH_2 ^{13}C-isotope	$C_1{}^{13}C_1H_6N_2O_1$	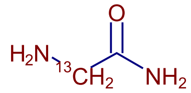	76.05>31.05<br>76.05>59.05	12<br>10	10<br>10
H_2N-Gly-Gly-NH_2 ^{13}C-isotope	$C_2{}^{13}C_2H_9N_3O_2$	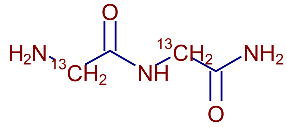	134.10>89.05<br>134.10>76.05<br>134.10>117.05	15<br>12<br>10	10<br>10<br>10
H_2N-Gly-Gly-Gly-NH_2 ^{12}C-isotope	$C_6H_{12}N_4O_3$	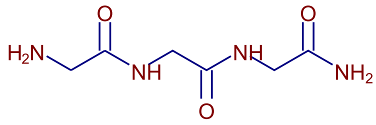	189.10>115.20<br>189.10>87.10<br>189.10>75.05<br>189.10>132.10<br>189.10>172.10	11<br>14<br>20<br>14<br>8	10<br>10<br>10<br>10<br>10
H_2N-Gly-Gly-Gly-NH_2 ^{13}C-isotope	$C_3{}^{13}C_3H_{12}N_4O_3$	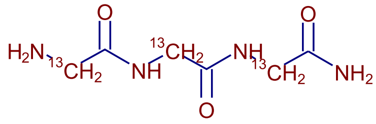	192.10>117.05<br>192.10>89.05<br>192.10>76.05<br>192.10>134.10<br>192.10>175.10	11<br>14<br>20<br>14<br>8	10<br>10<br>10<br>10<br>10
